# Nonsurgical Treatment of Radiculopathy From a Lumbar Synovial Cyst Using the Minimally Invasive Lumbar Decompression (MILD) Procedure: A Case Report

**DOI:** 10.7759/cureus.111194

**Published:** 2026-06-20

**Authors:** James V Lynch, Michael Verdolin

**Affiliations:** 1 Pain Management, Pain Consultants of San Diego, San Diego, USA

**Keywords:** fluoroscopy-guided procedure, interventional pain management, lateral recess stenosis, lumbar radiculopathy, lumbar spinal stenosis, lumbar synovial cyst, mild procedure, minimally invasive decompression

## Abstract

Lumbar synovial cysts are fluid-filled lesions arising from the facet joints that can cause lateral recess stenosis and neurogenic claudication. While surgical decompression is the gold standard for symptomatic cysts, patients with significant comorbidities, such as chronic obstructive pulmonary disease (COPD) and emphysema, face elevated perioperative risks under general anesthesia and delayed wound healing. This case report describes the successful application of the minimally invasive lumbar decompression (MILD) procedure as a safe alternative for a high-risk surgical candidate.

A 68-year-old male with a history of hypertension and severe COPD/emphysema presented with chronic, progressive lower back pain and fluctuating bilateral radiculopathy, which was initially worse on the left before migrating to the right and subsequently alternating sides. Lumbar MRI revealed a 6 mm × 5 mm synovial cyst arising from the left L4-L5 facet joint, causing severe subarticular narrowing and nerve root compression. To mitigate anesthetic risks, the patient underwent the MILD procedure under moderate sedation. Using a bone Kerrison and tissue sculptor, mechanical decompression of the lamina and hypertrophied ligamentum flavum was achieved, followed by an adjunct epidural injection of betamethasone.

The patient reported immediate symptomatic improvement. At the six-month follow-up, he maintained 100% relief of his primary radicular symptoms. A follow-up MRI confirmed successful decompression of the L4-L5 lateral recess and a significant reduction in cyst size. Although imaging later noted minimal residual tissue, the patient maintained sustained clinical improvement through March 2023 at his final follow-up.

The MILD procedure provides an effective alternative for symptomatic synovial cysts in patients who are poor candidates for open surgery. By combining mechanical decompression with anti-inflammatory measures under moderate sedation, clinicians can achieve sustained functional improvement while bypassing the respiratory risks associated with general anesthesia and invasive surgical excision.

## Introduction

Synovial cysts are fluid-filled sacs that may arise from joints throughout the body and, in the lumbar spine, most commonly originate from the facet joints [1]. Although typically benign, these cysts can cause symptoms similar to lumbar disc herniation, including numbness, burning pain, and lumbar radiculopathy due to nerve root impingement [2]. Standard treatment options include open surgical decompression or percutaneous aspiration [3,4]. However, open surgery may be highly invasive in elderly or medically complex patients, while aspiration alone is associated with high recurrence rates and limited long-term relief [2]. The minimally invasive lumbar decompression (MILD) procedure, a percutaneous technique for mechanical decompression of the lamina and ligamentum flavum, was used here as an off-label, minimally invasive alternative to traditional surgery, successfully managing the patient’s radicular symptoms [3,5].

## Case presentation

A 68-year-old male with a history of hypertension and severe chronic obstructive pulmonary disease (COPD)/emphysema presented with chronic, progressive lower back pain and fluctuating bilateral radiculopathy. The pain was characterized by numbness and tingling in the right calf that worsened with standing and walking. While the patient's dominant symptoms were right-sided, the MRI localized the synovial cyst to the left L4-L5 facet joint. This laterality mismatch is reconciled by clinical evidence that unilateral spinal pathology can manifest as contralateral radiculopathy due to traction forces on the nerve root rather than direct compression [6].

Physical examination revealed a flexed posture and gait consistent with neurogenic claudication, as well as reproducible pain upon facet loading. The patient’s baseline pain intensity was recorded as an 8/10 on the visual analog scale (VAS), a validated 10-point instrument where 0 represents no pain and 10 represents the most severe pain imaginable [7].

Lumbar MRI demonstrated multilevel foraminal stenosis and a 6 mm × 5 mm synovial cyst arising from the left L4-L5 facet joint. The cyst was positioned in the medial aspect of the lateral recess, resulting in severe subarticular narrowing and direct compression of the traversing L5 nerve root (Figure 1). The patient had failed conservative management, including interlaminar epidural steroid injections (ESIs). Given the patient’s clinical presentation and the significant surgical risk associated with his COPD/emphysema, the decision was made to proceed with the MILD procedure.

**Figure 1:**
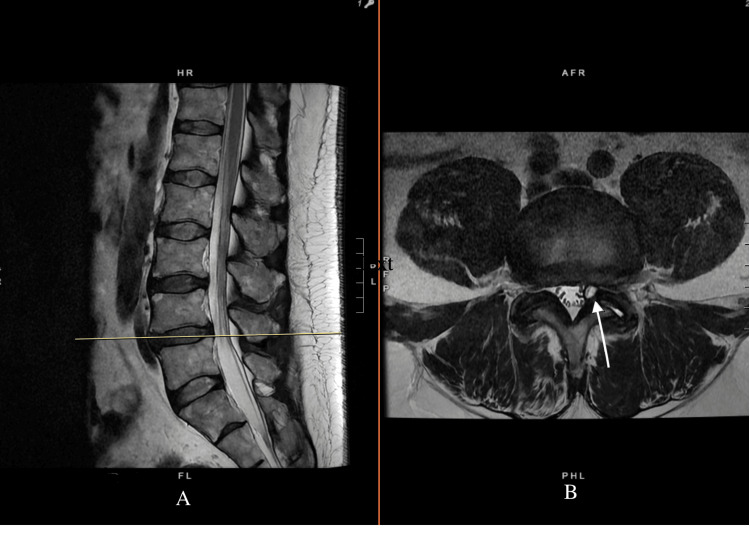
Preoperative T2-weighted axial and sagittal MRI of the lumbar spine (A) Sagittal view (left) and (B) axial view (right) demonstrate a large, well-circumscribed 6 mm × 5 mm synovial cyst arising from the medial aspect of the left L4-L5 facet joint. The cyst (white arrow) causes severe lateral recess stenosis and direct mechanical compression of the traversing L5 nerve root. HR: head/right; FL: foot/left; PHL: posterior/head/left; AFR: anterior/foot/right

To mitigate the elevated anesthetic risks associated with the patient’s COPD/emphysema, the procedure was performed under moderate intravenous (IV) sedation and local anesthesia rather than general anesthesia. With the patient in the prone position and under fluoroscopic guidance, the procedure was initiated by docking a 5.1 mm working cannula on the superior surface of the L5 lamina.

Using a bone Kerrison and tissue sculptor, portions of the lamina and hypertrophied ligamentum flavum were removed. The working cannula was docked on the superior surface of the L5 lamina with a trajectory aimed toward the left lateral recess to target the synovial cyst. Mechanical decompression was performed using the tissue sculptor to resect the hypertrophied ligamentum flavum, with the procedure endpoint determined by the tactile feedback of tissue clearance and the positioning of the instruments under continuous fluoroscopic guidance. This allowed for near-complete decompression of the synovial cyst along with targeted bone removal. Following the decompression, an epidural injection of 6 mg betamethasone and 1% lidocaine was administered at the L4-L5 level to address residual inflammation and mitigate the risk of cyst recurrence.

The patient reported immediate symptomatic improvement following the procedure. At the six-month post-procedure follow-up (March 2023), the patient reported 100% relief of his primary radicular symptoms. However, at that six-month appointment, the patient noted a gradual return of right lower-extremity pain, though it remained mild (VAS 2/10) and significantly improved from his initial baseline of 8/10.

A follow-up MRI in March 2023 confirmed the successful decompression of the L4-L5 lateral recess and a significant reduction in the size of the previously noted synovial cyst. While the acute mass effect on the L5 nerve root was resolved, imaging noted minimal residual cyst wall tissue and stable multilevel degenerative changes (Figure 2). This correlation between the objective reduction of the cyst and the patient's sustained symptomatic improvement (maintaining a VAS score of 2/10) highlights the efficacy of the MILD procedure for targeted decompression in high-risk surgical candidates.

**Figure 2:**
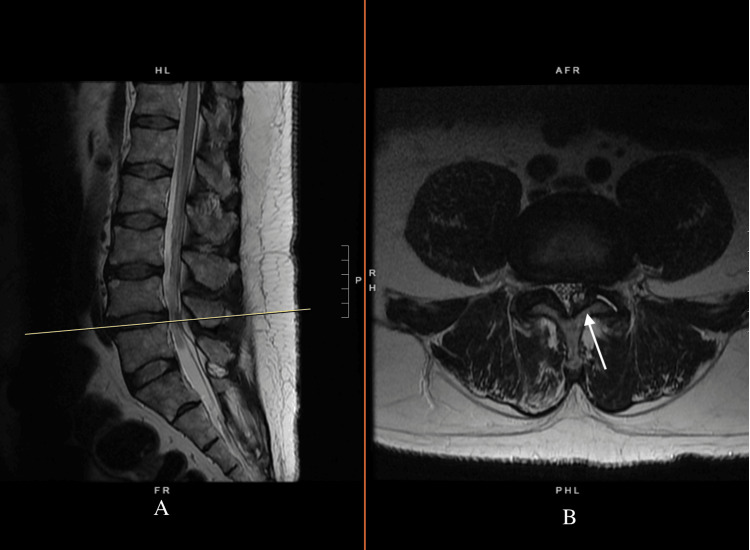
Postoperative T2-weighted MRI of the lumbar spine at six-month follow-up Follow-up imaging: (A) sagittal view (left) and (B) axial view (right) demonstrate successful decompression of the left L4-L5 lateral recess. There is a significant reduction in the size of the previously noted synovial cyst (white arrow), with resolution of the mass effect on the traversing L5 nerve root. HL: head/left; FR: foot/right; PHL: posterior/head/left; AFR: anterior/foot/right

## Discussion

Lumbar synovial cysts frequently present a therapeutic challenge, particularly in elderly patients with significant comorbidities who wish to avoid the perioperative risks associated with open surgery. In this patient, the presence of COPD and emphysema created a high-risk profile for general anesthesia, including potential difficulty with post-operative weaning and respiratory complications. Furthermore, patients with chronic respiratory illness and impaired systemic perfusion often exhibit delayed wound healing and an increased susceptibility to surgical site infections following invasive open procedures [8].

This case demonstrates that the MILD procedure effectively bridges the therapeutic gap for high-risk candidates. While the procedure is traditionally indicated for lumbar spinal stenosis caused by ligamentum flavum hypertrophy, our results suggest it is also a viable option for targeted decompression of synovial cyst-associated lateral recess stenosis [4]. The use of moderate IV sedation rather than general anesthesia was a critical safety component, allowing for successful mechanical decompression without compromising the patient's pulmonary status [9].

Furthermore, the administration of betamethasone following mechanical decompression helped mitigate the local inflammatory response and post-operative radiculitis. While percutaneous steroid injection and distention have been shown to be effective as primary interventions for synovial cysts, in this case, it was used as an adjunct to mechanical decompression to address inflammation [10].

Although the March 2023 follow-up MRI noted minimal residual tissue, the patient maintained a sustained VAS score of 2/10, highlighting that clinical success in these complex cases is defined by functional improvement and pain reduction rather than absolute radiological resolution.

## Conclusions

The MILD procedure offers a safe and effective alternative for patients with symptomatic lumbar synovial cysts, particularly those with significant comorbidities such as COPD and emphysema that may increase the risks associated with traditional open surgery under general anesthesia. By combining mechanical decompression with the anti-inflammatory effects of adjunctive betamethasone, this multimodal approach targets both the structural and inflammatory contributors to radiculopathy while avoiding the respiratory risks associated with intubation and postoperative ventilatory support. This case demonstrates that sustained clinical improvement, including marked pain reduction and improved function, may be achieved without the need for invasive surgical excision.
